# Robot‐Assisted Hip Arthroscopy for the Treatment of Femoroacetabular Impingement Syndrome Combined With Labral Injury: Surgical Technique

**DOI:** 10.1002/atn2.70120

**Published:** 2026-07-01

**Authors:** Xinyue Zhang, Zhe Xue, Ceyong Sun, Jin Zhang

**Affiliations:** ^1^ Department of Sports Medicine, Beijing Jishuitan Hospital Capital Medical University Beijing China; ^2^ Department of Sports Injury and Arthroscopic Surgery National Institute of Sports Medicine Beijing China; ^3^ Department of Orthopedics, Huairou Hospital of Beijing Chaoyang Hospital Capital Medical University Beijing China

## Abstract

Femoroacetabular impingement syndrome (FAIS) is one of the common causes of hip pain, limited range of motion, and labral injury. Without timely intervention, it may progress to hip osteoarthritis. In this study, a robot‐assisted hip arthroscopy technique was adopted, which involves preoperative bony structure image reconstruction, intraoperative real‐time navigation and positioning, and robotic arm‐assisted anchor placement. This technique enables hip labral repair and osteochondroplasty, restores normal acetabular‐femoral anatomy, and effectively addresses the shortcomings of traditional hip arthroscopy, such as insufficient precision in anchor positioning, angle, and depth, as well as excessive or inadequate osteophyte resection. It provides a more accurate and minimally invasive surgical option for patients with femoroacetabular impingement syndrome combined with labral injury.

## Graphical abstract

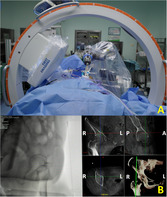

**VIDEO 1:** Robot‐assisted hip arthroscopy for the treatment of femoroacetabular impingement syndrome combined with labral injury: Surgical technique. The patient is supine. The video demonstrates: (1) Preoperative imaging diagnoses anterolateral overcoverage and labral tear of the right hip; (2) intraoperative setup with optical tracker fixation and robotic arm registration; (3) computer‐assisted planning for anchor placement; (4) robotic‐guided drilling and placement of suture anchors at the acetabular rim using a 2.5/3.0‐mm drill guide system; (5) arthroscopic view (anterolateral portal) of labral repair with vertical mattress sutures and femoral osteochondroplasty; and (6) postoperative imaging confirming impingement resection and accurate anchor positioning. Video content can be viewed at https://doi.org/10.1002/atn2.70120.

Femoroacetabular impingement syndrome (FAIS) with labral tear is a major cause of hip pain, often accompanied by chondral damage of the acetabular and femoral head, which is closely associated with the early onset and progression of hip osteoarthritis.^1^ The diagnosis of FAIS is primarily based on 3 criteria:^2^ (1) Pain in the groin or the “C‐sign” area on the affected side; (2) positive anterior impingement test (which can be enhanced by internal or external rotation), positive FABER test, and negative Ober's test; (3) magnetic resonance imaging of the hip shows a high‐grade (grade III) signal intensity at the labrum, and 3‐dimensional computed tomography (3D CT) reveals impingement morphology (pincer or cam deformity) anterolaterally.

Traditional hip arthroscopy surgery relies on the surgeon's experience for anchor placement, labral repair, and osteochondroplasty. However, due to the deep and complex anatomy of the hip, the surgeon often faces limited visualization and a confined operating space. This frequently leads to insufficient precision in anchor positioning, angle, and depth, as well as excessive or inadequate osteophyte resection, potentially resulting in complications like intraoperative anchor placement into the hip joint or pelvic cavity, persistent postoperative impingement, recurrent labral tear, or femoral neck fracture.^3^, ^4^, ^5^, ^6^, ^7^, ^8^ Consequently, hip arthroscopy is one of the more challenging procedures in sports medicine with a steep learning curve. In recent years, the application of computer‐assisted technology has provided surgeons with new concepts and techniques that may significantly enhance surgical precision and intraoperative safety, thereby reducing the incidence of postoperative complications.^9^, ^10^, ^11^, ^12^, ^13^ Kobayashi et al.^14^ summarized the standardized workflow of computer‐assisted hip arthroscopy for FAIS: preoperative simulation assessment to accurately locate the osteophyte hyperplasia; virtual surgical planning for femoroacetabular osteoplasty; and finally, intraoperative real‐time navigation.

Therefore, based on the standardized workflow proposed by Kobayashi and the work of Thomas et al.^6^ on computer‐assisted fluoroscopic navigation for cam resection, this study utilized a robot‐assisted hip arthroscopy system (TINAVI Tianji II Orthopedic Surgical Robot). Through preoperative 3D reconstruction of bony structures, intraoperative navigation, and robotic arm‐assisted anchor placement, the complete surgical procedure was performed for a patient with FAIS combined with labral injury (Video 1).

## SURGICAL TECHNIQUE

### Preoperative Modeling

**FIGURE 1:**
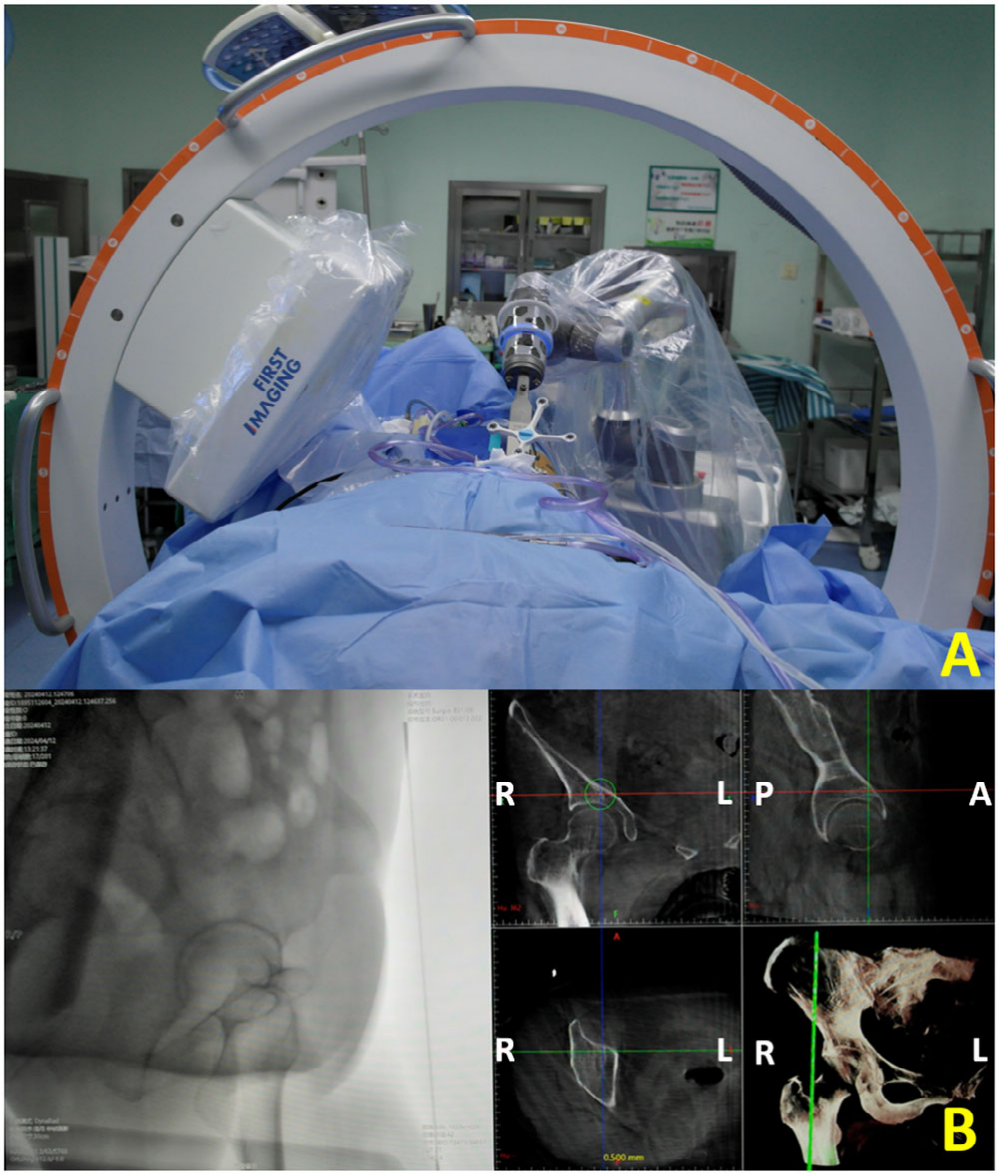
Preoperative bony structure image reconstruction. (A) The patient was placed in the supine position, and 3‐dimensional (3D) computed tomography scan of the affected right hip. (B) Reconstructed images of the affected right hip (oblique coronal view, oblique sagittal view, axial view, and 3D reconstruction, respectively).

**FIGURE 2:**
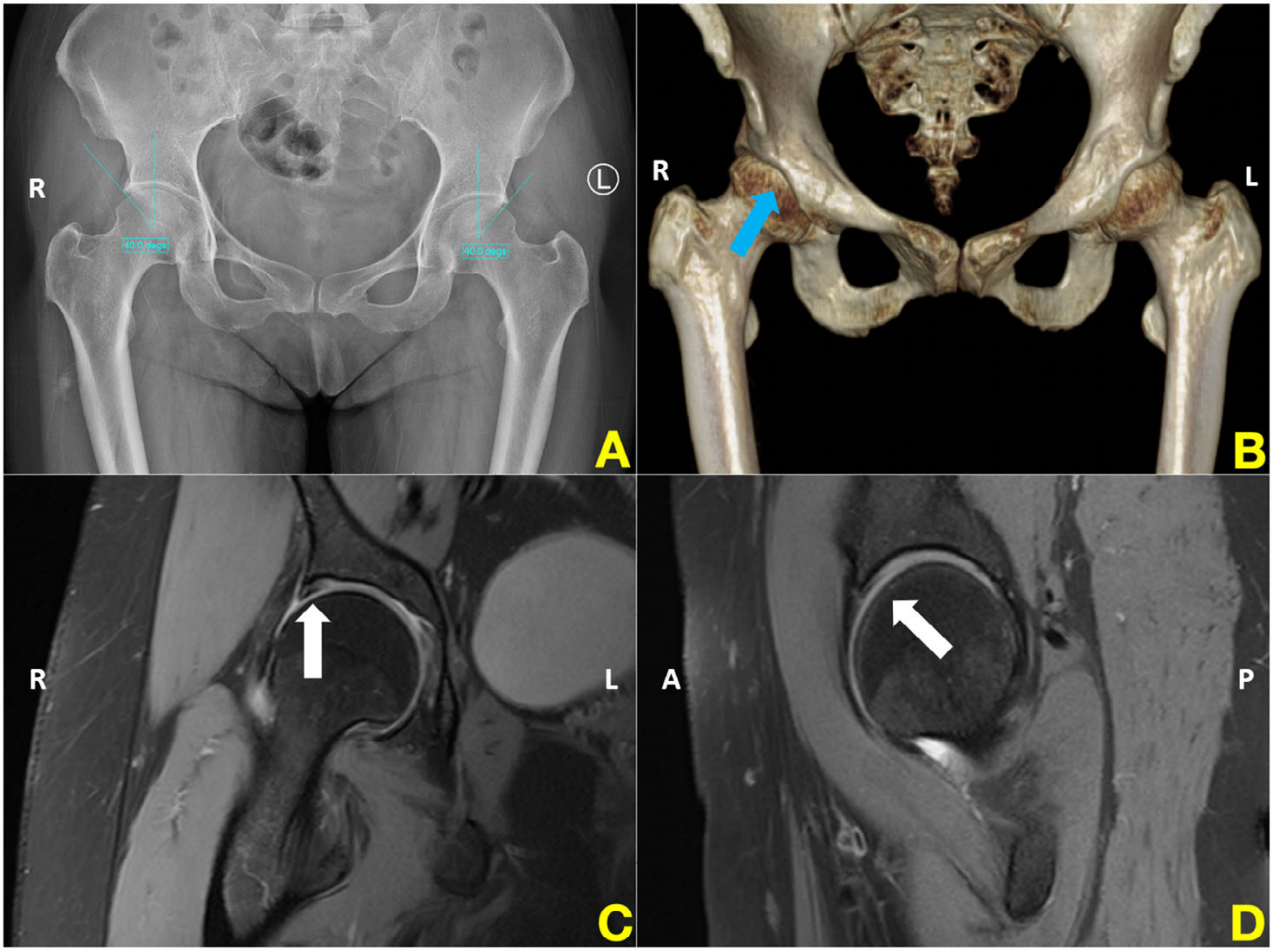
Preoperative imaging examinations. (A) Weight‐bearing pelvic anteroposterior radiograph, showing the lateral center‐edge angle of the right hip is 40°; (B) 3‐dimensional computed tomography reconstruction image, showing excessive coverage of the anterior and lateral acetabulum of the right hip with osteophyte hyperplasia (blue arrow); (C,D) magnetic resonance imaging oblique coronal and oblique sagittal views, showing obvious labral tear in the right hip (white arrow).

Preoperatively, with the patient in the conventional supine position and the affected limb under traction, a corresponding CT scanner is used to perform a 360° scan of the affected hip to construct a 3D bony model (Figure 1). Weight‐bearing anteroposterior pelvic radiographic examination and 3D CT reconstruction show anterior and lateral acetabular overcoverage with pincer and cam‐type osteophyte hyperplasia, and the lateral center‐edge angle is measured at 40°. Magnetic resonance imaging shows an obvious labral tear in the right hip on oblique sagittal and oblique coronal views, accompanied by partial chondral damage (Figure 2).

### Intraoperative Fine Registration

**FIGURE 3:**
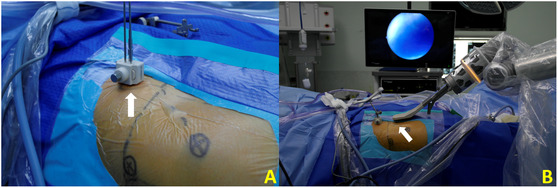
Intraoperative fine registration. (A) With the patient in the supine position, an optical tracker is fixed to the anterior superior iliac spine of the right hip. (B) Fine registration of the robotic arm (TINAVI Tianji II) for the right hip.

The patient is placed in a supine position, and an optical tracker is securely fixed to the bony prominence of the anterior superior iliac spine (Figure 3A) to ensure stability of intraoperative positioning. The robotic arm, connected to a calibrator, is brought close to the surgical area (Figure 3B), and another 360° CT scan is performed to acquire images, which are transmitted to the computer host, processed, and displayed on the monitor.

### Anchor Placement Planning

**FIGURE 4:**
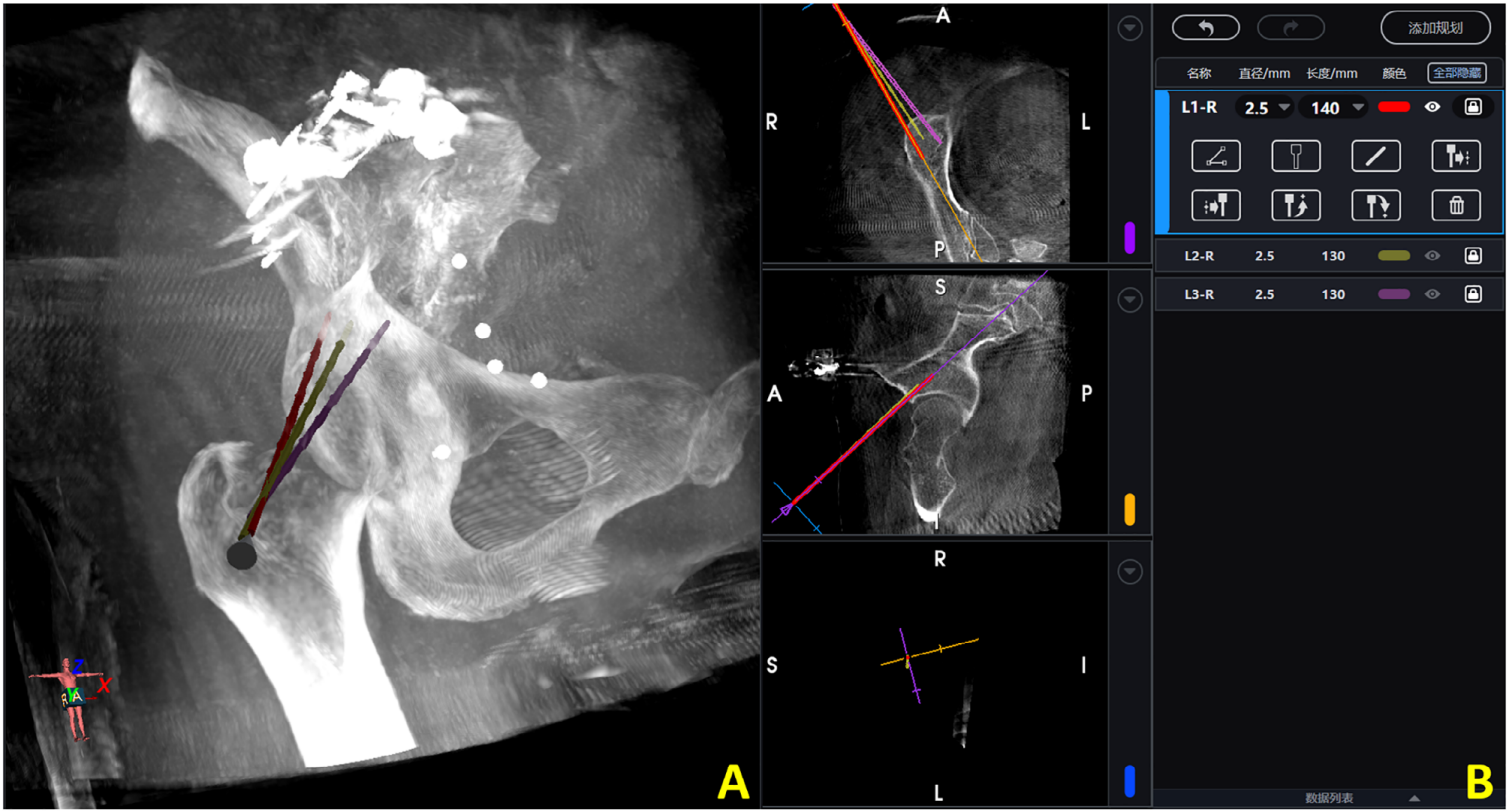
Intraoperative fine registration and anchor placement planning under robot assistance for the right hip. (A) The red, green, and purple lines represent the details of the 3 planned anchors and (B) confirmation of satisfactory anchor position using the registered sagittal, coronal, and axial images.

Using the finely registered 3D model reconstructed by the computer, the intraoperative anchor placement plan is designed, and precise operation commands are sent to the robot host: based on the morphology and location of the pincer bony deformity, the planned positions, directions, and depths for 3 anchors are drawn, as shown in Figure 4A, where the red, green, and purple lines represent the details of the 3 planned anchors; Figure 4B shows confirmation of satisfactory anchor positioning using the registered sagittal, coronal, and axial images.

### Robot‐Assisted Anchor Placement

**FIGURE 5:**
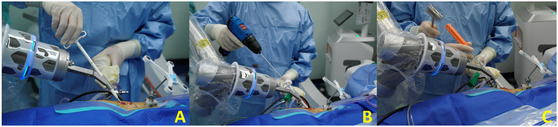
Robot‐assisted anchor placement for the right hip. (A) Installation of a 2.5 mm diameter guide on the robotic arm (TINAVI Tianji II); (B) replacement of the guide with a 2.4 mm one and creation of a tunnel along its direction; (C) replacement of the guide with a 3.0 mm one and insertion of a PEEK suture anchor (Youwin, China) into the tunnel.

Under fluoroscopic guidance, a standard anterolateral portal is established to access the hip. Through the mid‐anterior portal, the capsule is incised from outside‐in using low‐temperature plasma radiofrequency. After completing coarse registration (controlled by the black pedal) and fine registration (controlled by the yellow pedal) sequentially, a drill guide (initially 2.5 mm diameter to minimize “windshield wiper effect”, Figure 5A) is attached to the robotic arm (TINAVI Tianji II). According to the planned anchor positions, anchors are precisely placed at the site of the acetabular pincer deformity after osteoplasty (3 anchors planned). A 2.4 mm diameter depth‐limited drill is then used to prepare the tunnel, with a planned tunnel depth of 15 mm (anchor length 14.4 mm, Figure 5B). With the robotic arm position fixed, the guide is changed to a 3.0 mm diameter, and a 3.0 mm PEEK suture anchor (Youwin, China) is inserted along the guide into the tunnel and impacted for press‐fit fixation (Figure 5C).

### Labral Repair and Head‐Neck Junction Osteochondroplasty

**FIGURE 6:**
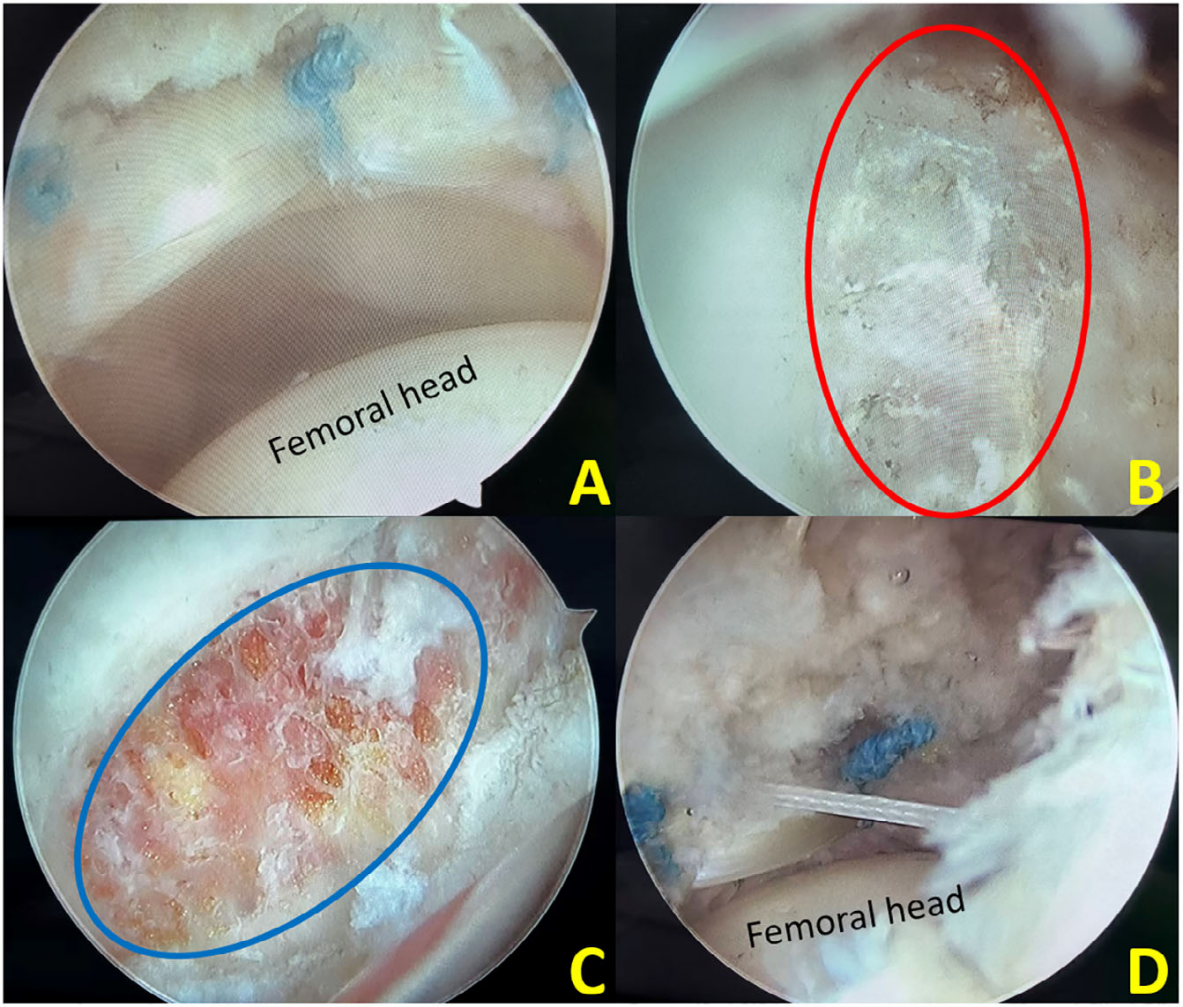
Labral suture and osteophyte debridement of the right hip, viewed from the anterolateral portal. (A) 3 vertical mattress sutures for the torn labrum; (B) hyperplastic osteophytes (red circle); (C) femoral head‐neck junction after osteophyte debridement; (D) suture of the joint capsule using the capsular suture‐lifting technique.

The anterolateral portal serves as the viewing portal, and the mid‐anterior portal is used as the working portal. Using a curved suture passer introduced through the mid‐anterior portal, the vertical mattress suture technique is performed with the sutures from the placed PEEK anchors (Youwin, China). The sutures are then tensioned and secured with a Tennessee sliding knot to restore labral integrity and tension (Figure 6A). A bone burr was used to precisely perform osteochondroplasty on the bony prominences at the femoral head‐neck junction, eliminating the femoroacetabular impingement factors (Figure 6B and C). The capsule is repaired using the capsular suture‐lifting technique (Figure 6D).

### Postoperative Imaging

**FIGURE 7:**
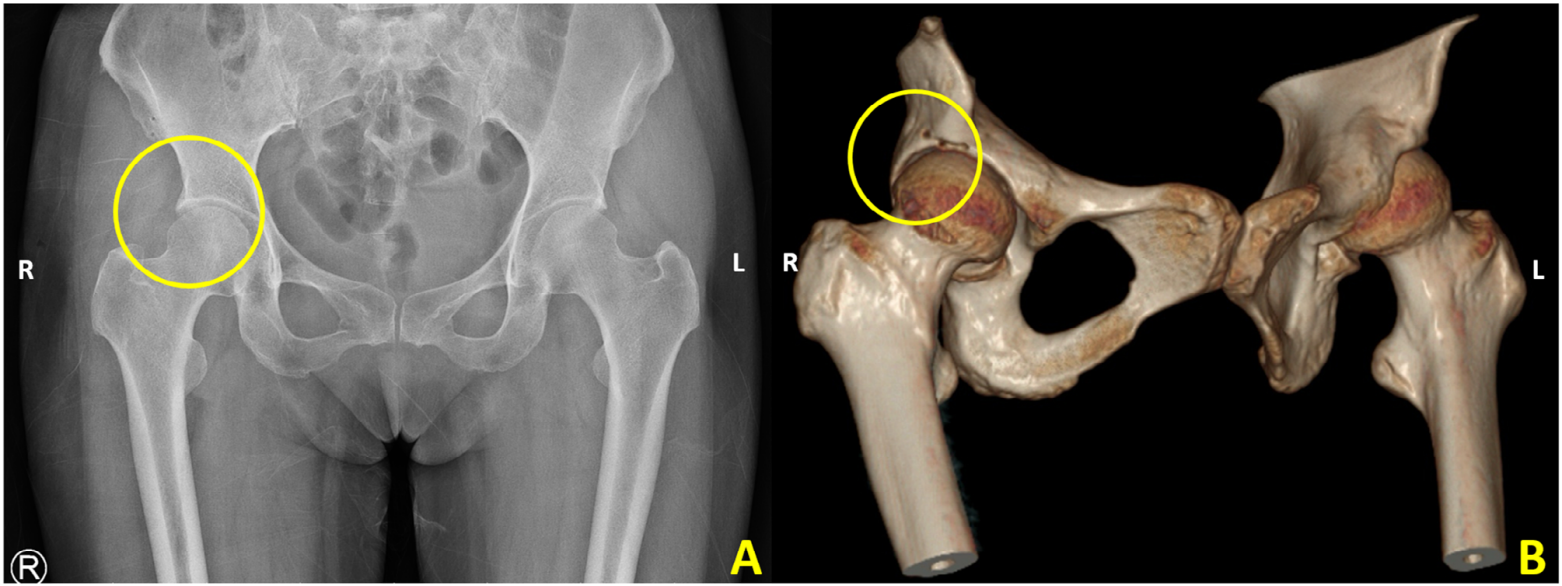
Postoperative imaging examinations. (A) Weight‐bearing pelvic anteroposterior radiograph, showing elimination of the right hip impingement structure; (B) 3‐dimensional computed tomography reconstruction of the hip, showing good anchor position in the right hip, consistent with the intraoperative plan.

Radiograph and CT re‐examinations on the first postoperative day shows that the hip impingement structure is completely eliminated, the anchor tunnel on the acetabular side is in a good position, and the tunnel position is completely consistent with the intraoperative plan (Figure 7).

## DISCUSSION

In this case, robot‐assisted hip arthroscopy was used to treat a patient with FAIS combined with labral injury. The surgery was successfully completed through preoperative bony structure image reconstruction, intraoperative fine registration, navigation and positioning, and robotic arm‐assisted anchor placement. The key points and precautions of the surgery are summarized in Table 1.

**TABLE 1:** Pearls and Pitfalls

Pearls	Pitfalls
Preoperative 3‐dimensional (3D) bony structure image reconstruction	Loosening of the tracker can cause navigation deviation
Fine registration for bony model reconstruction	Maintain a stable body position; otherwise, registration accuracy will decrease
Robotic arm‐assisted tunnel creation and anchor placement	First perform manual coarse registration (black pedal), then automatic fine registration (yellow pedal) before anchor placement

A systematic review on computer‐assisted hip arthroscopy indicates that this technology can provide more precise surgical planning, reduce human error, and improve patient outcomes.^15^ Looney et al. and Gursoy et al.^16^, ^17^ used computer vision interfaces to measure and compare the alpha angle on intraoperative fluoroscopic images before and after bone resection in real‐time. Stražar^5^ and Kobayashi et al.^14^ combined preoperative planning software with intraoperative navigation systems for kinematic planning of the resection area and real‐time tracking of surgical instruments displaying resection progress. Park et al.^18^ used the MAKO Robotic arm Interactive Orthopedic System (RIO) (MAKO Surgical, Fort Lauderdale, FL) to assist with femoral osteoplasty, providing visual and haptic feedback to surgeons. Kather et al.^19^ applied the da Vinci Surgical System to hip arthroscopy. Masayoshi et al.^20^ performed computer navigation‐assisted osteochondroplasty and showed that it may improve the accuracy of cam resection and contribute to better short‐term outcomes. Higashihira et al.^21^ utilized preoperative 3D CT‐based dynamic simulation for virtual osteochondroplasty planning, performing computer‐assisted hip arthroscopy for secondary FAIS after rotational acetabular osteotomy. Different from previous studies that primarily used robotic navigation software alone, this technique used a complete robot system and robotic arm to perform preoperative 3D bony structure reconstruction, intraoperative tunnel localization, and assisted anchor placement. In contrast, previous studies involved robotic navigation followed by manual anchor placement, which distinguishes this technique from prior methods.

Due to the complex anatomy, deep location, and dense surrounding neurovascular structures of the hip joint, surgical procedures are highly challenging and technically demanding. Previous open surgeries often resulted in significant trauma and complications,^22^, ^23^, ^24^ making hip arthroscopy the current preferred approach for treating hip disorders.^3^ Although hip arthroscopy for FAIS can achieve good outcomes,^25^ the technique has a long learning curve. Traditional arthroscopy relies more on the surgeon's extensive clinical experience and is prone to issues like under‐ or over‐resection of bone, leading to impingement recurrence or risk of femoral neck fracture.^6^, ^26^, ^27^ Therefore, the application of this technology provides a more accurate and stable operating environment for hip arthroscopy. Through preoperative image reconstruction and intraoperative real‐time feedback, it reduces operative errors, improves surgical success rates, and lowers the incidence of intraoperative and postoperative complications.^9^, ^10^

The advantage of this surgical technique lies in the robot‐assisted preparation of tunnels and anchor placement, resulting in more precise anchor insertion. This avoids labral repair instability or impingement due to anchor placement deviations in traditional surgery, reduces secondary damage to articular cartilage, and significantly improves surgical precision and safety. Furthermore, optical tracking and robotic arm assistance in this technique can greatly reduce intraoperative fluoroscopy, lowering radiation exposure for both doctors and patients, and shortens the learning curve, enabling less experienced surgeons to perform precise operations.^28^, ^29^, ^30^, ^31^ However, this technique still has certain limitations. In this surgery, the osteochondroplasty at the femoral head‐neck junction was not performed with robotic assistance and relied on the surgeon's experience, potentially affecting the precision of bone resection. Additionally, the technique faces challenges such as high equipment costs and long preoperative planning time. Future development should focus on developing dedicated software modules and hardware equipment, optimizing the robotic system workflow to enable precise robotic‐assisted osteochondroplasty and reduce medical costs. Table 2 summarizes the advantages and disadvantages of robot‐assisted surgery.

**TABLE 2:** Advantages and Disadvantages of Robot‐Assisted Surgical Technique

Advantages	Disadvantages
High surgical precision, avoiding complications from anchor placement deviation	High equipment costs
Intraoperative real‐time monitoring	Time‐consuming preoperative planning
Reduced intraoperative fluoroscopy, lower radiation exposure	Additional skin incision required for optical tracker placement
Reduced reliance on experience, assists less experienced surgeons	Lack of training for arthroscopic tactile feedback
	Osteochondroplasty still relies on surgeon's experience, not fully robot‐assisted

In conclusion, robot‐assisted hip arthroscopy is effective in the treatment of FAIS combined with labral injury. This technique has the advantages of higher precision, minimal invasiveness, and greater safety, which can shorten the learning curve of hip arthroscopy, effectively restore hip anatomy and function, and reduce postoperative complications, providing a surgical technical option for the treatment of this disease.

## DISCLOSURES

The authors (Z.X., J.Z.) declare the following financial interests/personal relationships which may be considered as potential competing interests: Z.X. reports financial support that was provided by National Natural Science Foundation of China. J.Z. reports financial support that was provided by Beijing Municipal Administration of Hospitals; Beijing Medical and Health Science and Technology Promotion Center; Beijing Association for Science and Technology; Beijing Jishuitan Hospital Affiliated to Capital Medical University; Beijing Nova Program; and Science and Technology Program of XPCC. The other authors (X.Z., C.S.) declare that they have no known competing financial interests or personal relationships that could have appeared to influence the work reported in this paper.
